# Urinary complement proteins are increased in children with IgA vasculitis (Henoch-Schönlein purpura) nephritis

**DOI:** 10.1007/s00467-022-05747-3

**Published:** 2022-10-13

**Authors:** Rachael D. Wright, Julien Marro, Sarah J. Northey, Rachel Corkhill, Michael W. Beresford, Louise Oni

**Affiliations:** 1 Department of Women’s and Children’s Health, Institute of Translational Medicine, University of Liverpool, Member of Liverpool Health Partners, Eaton Road, Liverpool, L12 2AP UK; 2 Department of Paediatric Rheumatology, Alder Hey Children’s NHS Foundation Trust, Member of Liverpool Health Partners, Liverpool, UK; 3 Department of Paediatric Nephrology, Alder Hey Children’s NHS Foundation Trust, Member of Liverpool Health Partners, Eaton Road, Liverpool, L12 2AP UK

**Keywords:** Nephritis, Vasculitis, Immunoglobulin A, Complement, Biomarker, Urine

## Abstract

**Background:**

Children with immunoglobulin A vasculitis (IgAV Henoch-Schönlein purpura) frequently encounter nephritis (IgAV-N) with 1–2% risk of kidney failure. The pathophysiology of IgAV-N is not fully understood with speculation that complement may contribute. The aim of this study was to identify whether urinary complement proteins are increased in children with IgAV-N.

**Methods:**

A cross-sectional prospective cohort of children with IgAV were recruited together with controls including healthy children and children with systemic lupus erythematosus (SLE). Patients were subdivided according to the presence of nephritis. Urinary C3, C4, C5, and C5a were measured by enzyme-linked immunosorbent assay (ELISA) and corrected for urinary creatinine.

**Results:**

The study included 103 children; 47 with IgAV (37 IgAV without nephritis, IgAVwoN; 10 IgAV-N), 30 SLE and 26 healthy children. Urinary complement C3, C4, and C5 were all statistically significantly increased in all children with IgAV compared to SLE patients (all *p* < 0.05). In patients with IgAV-N, urinary complement C3, C4, C5, C5a were all statistically significantly increased compared to IgAVwoN (C3 14.65 μg/mmol [2.26–20.21] vs. 2.26 μg/mmol [0.15–3.14], *p* = 0.007; C4 6.52 μg/mmol [1.30–9.72] vs. 1.37 μg/mmol [0.38–2.43], *p* = 0.04; C5 1.36 μg/mmol [0.65–2.85] vs. 0.38 μg/mmol [0.03–0.72], *p* = 0.005; C5a 101.9 ng/mmol [15.36–230.0] vs. 18.33 ng/mmol [4.27–33.30], *p* = 0.01). Using logistic regression, the urinary complement components produced an outstanding ability to discriminate between patients with and without nephritis in IgAV (AUC 0.92, *p* < 0.001).

**Conclusions:**

Children with IgAV-N have evidence of increased complement proteins present in their urine that may indicate a pathological role and may allow treatment stratification.

**Graphical abstract:**

**Figure:**
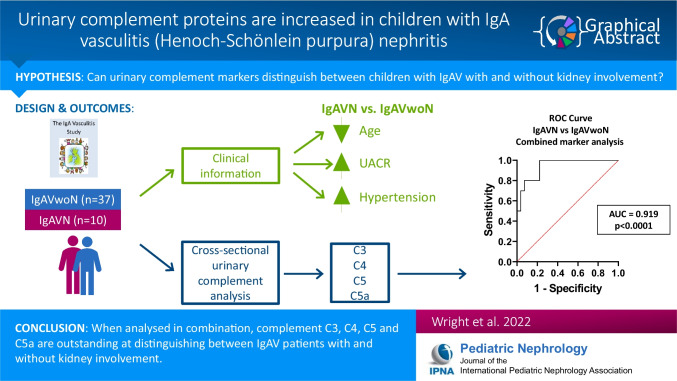
A higher resolution version of the Graphical abstract is available as Supplementary information

**Supplementary Information:**

The online version contains supplementary material available at 10.1007/s00467-022-05747-3.

## Introduction

Immunoglobulin A vasculitis (IgAV), formerly known as Henoch-Schönlein purpura, is the most common form of vasculitis in children with an estimated incidence of around 20 cases per 100,000 children per year. It is caused by deposition of aberrantly glycosylated IgA in tissues leading to activation of an autoimmune response [1]. IgAV is self-limiting in 50–70% of cases and most patients will make a complete and full recovery with no intervention. Kidney involvement (termed IgAV nephritis – IgAV-N) is the most damaging consequence of IgAV as it is the only organ affected that is associated with long-term morbidity and carries a 1–2% risk of progression to kidney failure [2, 3]. There are unmet needs in understanding the pathophysiology of IgAV-N and kidney outcomes have not demonstrated any improvement over time [4]. Histologically, glomerular IgA deposition is universally seen and in 90% of cases it is co-located with complement C3. There is growing interest in the role of the complement pathway in IgA-related kidney diseases [1, 5] and in patients with IgA nephropathy (IgAN), reports demonstrate elevated serum concentrations of C3d, C4d, C5b-9, mannose-binding lectin, and mannose-associated serine protease-1 [6–8], such that selective therapeutic inhibition of the lectin and alternative complement pathways are under current evaluation. In other proteinuric kidney diseases, complement activation products are reported to be increased in patients with focal glomerular sclerosis and diabetic nephropathy but not in heavy proteinuria associated with minimal change disease, suggesting that the findings may have an active pathological role in certain inflammatory kidney diseases [9]. In children with IgAV-N, there are limited corresponding data available, apart from histological evidence of glomerular complement deposition (C3, C4 and C5b-9) [10, 11].

The aim of this study was to identify whether urine complement proteins are present in children with IgAV, how they compare to another form of glomerulonephritis and whether they are able to distinguish patients with IgAV-N.

## Methods

### Study cohort

A cross-sectional cohort of patients was recruited for this study. A one-off urine sample was collected at the time of appointment from a cohort of patients with IgA vasculitis and healthy controls obtained as part of the IgA Vasculitis Study (REC: 17/NE/0390) and a cohort of children with SLE and healthy controls from participants within the UK JSLE Cohort Study and Repository (REC: 6/Q1502/77). Informed consent was obtained from all subjects, and if subjects were under the age of 16 years, consent was obtained from a parent and/or legal guardian and assent was obtained from the subject as appropriate. A cohort of age- and sex-matched healthy control participants were used for comparison. For data analysis purposes, healthy controls recruited as part of both studies were pooled as a single control population.

### Nephritis classification

Patients within the disease categories (IgAV and SLE) were subdivided according to the presence of nephritis. Nephritis in children with IgAV (termed IgAV-N group) was defined as a urinary albumin to creatinine ratio (ACR) > 30 mg/mmol Cr at the time of sampling. Patients with IgAV who had a normal urine dipstick and/or a urine ACR < 30 mg/mmol Cr at the time of sampling were considered not to have any significant nephritis (termed IgAVwoN). Nephritis in patients with SLE was defined according to the British Isles Lupus Assessment Group (BILAG) 2004 index [12, 13]. Patients with a BILAG score of A or B in their kidney domain (new or worsening hypertension, proteinuria, reduced kidney function, nephrotic syndrome, active urinary sediment and/or active histological evidence of nephritis) were considered to have had nephritis (lupus nephritis, termed LN group) while those with a BILAG score of E in their kidney domain (no previous evidence of nephritis; termed SLEwoLN) were considered not to have LN.

### Laboratory methods

Enzyme-linked immunosorbent assay (ELISA) kits were purchased to detect C3, C4, C5, and C5a (Bio-Techne, Abingdon, UK) and were run per the manufacturer’s instructions. Briefly, urine samples were collected as a clean catch sample and immediately transferred to the onsite laboratory for processing. They were spun at 300 g for 5 min to remove any particulate matter and loaded neat onto plates to analyse levels of complement protein in each sample. Urine infection was excluded in all samples prior to analysis either through a urine dipstick that was negative for nitrites and leucocytes or through formal microbiological examination. All urine complement concentrations were corrected for concentration using urinary creatinine measured by the Clinical Chemistry Laboratory at Alder Hey Children's NHS Foundation Trust using the Abbott Enzymatic Creatinine assay on the Abbott Architect Ci8200 (Abbott, Illinois, USA).

### Statistical analysis

Urinary complement levels were normalised to urinary creatinine in all instances. Data are expressed as median [range] unless otherwise stated. Statistical analysis was performed using GraphPad Prism 7.01 software programme to compare between the disease cohorts IgAV, SLE and healthy controls and between the subgroups with nephritis (IgAVwoN, IgAV-N, SLEwoLN, LN, healthy controls). Data normality was assessed using Shapiro–Wilk test. Statistical significance was evaluated using Kruskal–Wallis test with Dunn’s post hoc test. Univariate and logistic regression analyses were performed, and receiver operating characteristic (ROC) curves were generated to evaluate the ability of each urinary complement protein to predict the patients with nephritis. The area under the curve (AUC) value was used to assess the strength of the test in distinguishing patients with IgAV-N and defined accordingly (AUC 0.7–0.8 considered acceptable, 0.8–0.9 considered excellent, 0.9–1.0 considered outstanding). A *p* value of less than 0.05 was considered statistically significant.

## Results

### Patient demographic data and baseline characteristics

The study included 103 children, consisting of 77 patients with an inflammatory disease and 26 participants who were age- and sex-matched healthy controls. A total of 47 children with IgAV contributed and of these 37 participants were grouped as IgAVwoN and 10 participants were classed as IgAV-N. There were 30 children with SLE consisting of 15 SLE participants without any history of ever having had nephritis (SLEwoLN) and 15 with a history of LN. The demographic data and baseline characteristics are presented in Table 1. As expected, there was an increased proportion of male patients with IgAV and an increased proportion of female patients with SLE, consistent with the overall gender distribution seen for these conditions. SLE patients were significantly older than those with IgAV and healthy controls, consistent with the expected overall age distribution of these conditions. The healthy control cohort was reasonably well-matched in terms of demographics. Patients with IgAV-N had a significantly increased urinary albumin-to-creatinine ratio (UACR) compared to IgAVwoN, SLEwoLN, and LN. Similar types of immunosuppressant therapies were used within the IgAV and SLE cohorts; however, as expected, these were used more frequently in patients with SLE.

**Table 1:**
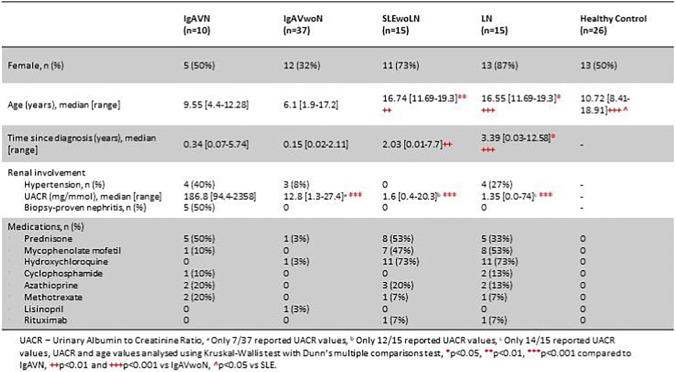
Demographic and baseline data for patients included in this study

### Urinary complement concentration in all patients with IgAV

**Fig. 1:**
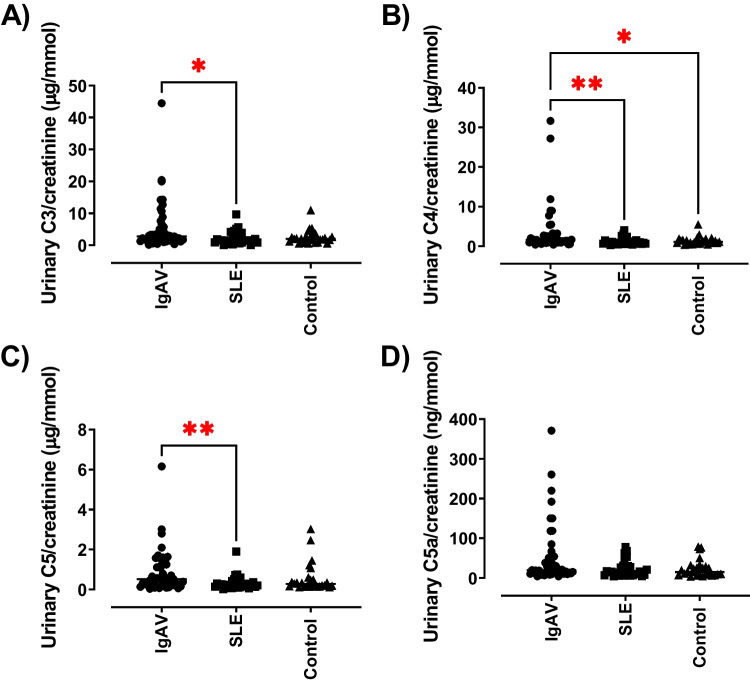
Urinary complement concentrations in patients with paediatric inflammatory disease – IgAV and SLE compared to age- and sex-matched controls. Complement concentrations were assessed in urine collected from patients with IgAV, SLE and controls using ELISA. Complement concentrations were normalised to urinary creatinine. (**A**) Urinary C3/creatinine, (**B**) Urinary C4/creatinine, (**C**) Urinary C5/creatinine, and (**D**) Urinary C5a/creatinine. N = 26–47/group. Data are expressed as median and analysed using Kruskal–Wallis test with Dunn’s multiple comparison test. *P < 0.05 and **P < 0.01

Urinary complement concentrations (C3, C4, C5 and C5a) were assessed in all patients with IgAV and compared to patients with SLE and the healthy control group. The urinary complement C3 concentration was statistically significantly increased in all patients with IgAV compared to patients with SLE (median: IgAV 2.74 μg/mmol [0.15–44.5], SLE 1.52 μg/mmol [0.09–9.66]); *p* = 0.021), as shown in Fig. 1A. There was no statistically significant difference in the urinary C3 concentrations between patients with IgAV and healthy controls (1.98 μg/mmol [0.49–11.0]). Urinary complement C4 concentrations were also statistically significantly increased in patients with IgAV (median: 1.56 μg/mmol [0.38–31.66]) compared to those with SLE (0.87 μg/mmol [0.25–4.01]; *p* = 0.001) and with the healthy control participants (1.08 μg/mmol [0.37–5.46]; *p* = 0.03) (Fig. 1B). There were no statistically significant differences seen between SLE patients and healthy control participants. The urinary complement C5 concentrations were statistically significantly increased in IgAV patients (median: 0.51 μg/mmol [0.03–6.16]) compared to patients with SLE (0.20 μg/mmol [0.02–1.90]; *p* = 0.008) (Fig. 1C). No statistically significant differences were seen between patients with IgAV and healthy control participants (0.28 μg/mmol [0.10–3.02]) or between patients with SLE and healthy controls. Urinary complement C5a was not statistically significantly different between any of the groups. However, there was a trend toward increased levels in the IgAV group (19.69 ng/mmol [4.27–370.9]) to SLE (14.32 ng/mmol [3.92–78.11]; *p* = 0.06) (Fig. 1D).

### Urinary complement concentration in patients with IgAV nephritis

**Fig. 2:**
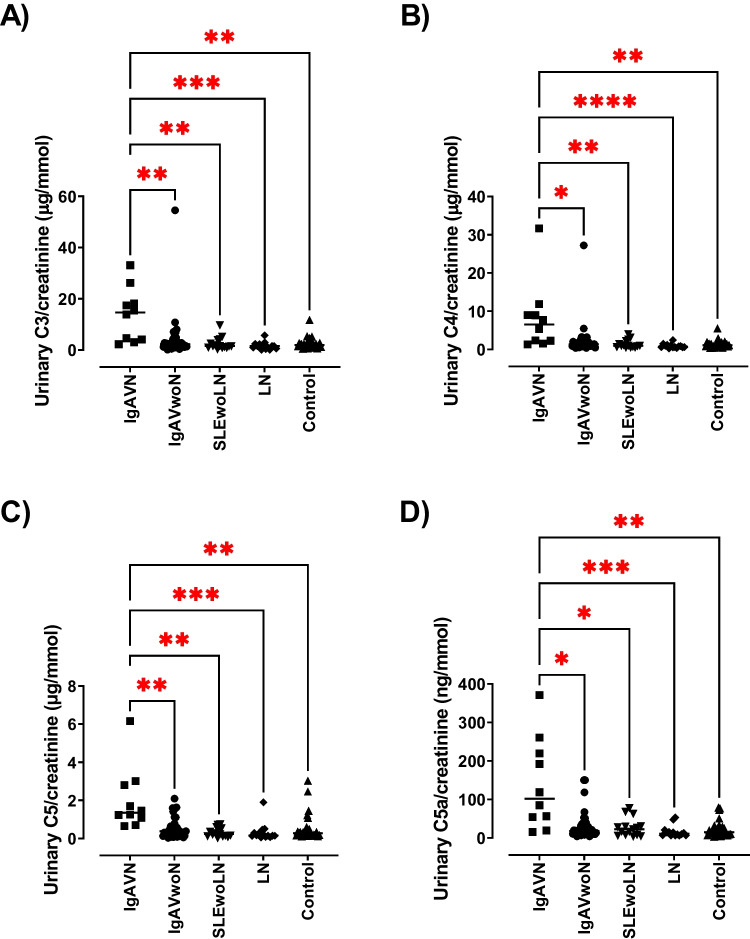
Urinary complement concentrations in patients with paediatric inflammatory disease stratified by kidney involvement – IgAVwoN, IgAV-N, SLEwoLN and LN compared to age- and sex-matched controls. Complement concentrations were assessed in urine collected from patients with IgAVwoN, IgAV-N, SLEwoLN, LN and controls using ELISA. Complement concentrations were normalised to urinary creatinine. (**A**) Urinary C3/creatinine, (**B**) Urinary C4/creatinine, (**C**) Urinary C5/creatinine, and (**D**) Urinary C5a/creatinine. N = 10–37/group. Data are expressed as median and analysed using Kruskal–Wallis test with Dunn’s multiple comparison test. *P < 0.05, **P < 0.01, ***P < 0.001 and ****P < 0.0001

The participants were subdivided according to the presence of nephritis. The concentration of urinary complement proteins (C3, C4, C5 and C5a) was evaluated to see if these could distinguish patients with and without nephritis in IgAV. The urinary complement C3 concentration was statistically significantly increased in patients with IgAV-N (median: 14.65 μg/mmol [2.26–20.21]) compared to patients grouped as IgAVwoN (2.26 μg/mmol [0.15–3.14]; *p* = 0.007) (Fig. 2A). They were also statistically significantly increased in IgAV-N compared to patients with LN (1.52 μg/mmol [0.54–2.27]; *p* = 0.0002). The urinary complement C3 concentration was also statistically significantly increased in patients with IgAV-N compared to healthy controls (median: 1.89 μg/mmol [0.49–2.94]; *p* = 0.003). No other significant differences between the groups were noted. The urinary complement C4 concentration was statistically significantly increased in IgAV-N patients (6.52 μg/mmol [1.30–9.72]) compared to IgAVwoN (1.37 μg/mmol [0.38–2.43]; *p* = 0.04), and compared to all other groups – SLEwoLN (0.98 μg/mmol [0.47–2.23]; *p* = 0.007), LN (0.72 μg/mmol [0.25–1.12]; *p* < 0.0001) and healthy controls (1.08 μg/mmol [0.37–1.57]; *p* = 0.0012) (Fig. 2B). No other significant differences between groups were noted. Urinary complement C5 concentration was statistically significantly increased in IgAV-N patients (1.36 μg/mmol [0.65–2.85]) compared to IgAVwoN (0.38 μg/mmol [0.03–0.72]; *p* = 0.005) and all other groups – SLEwoLN (0.24 μg/mmol [0.02–0.55]; *p* = 0.001), LN (0.16 μg/mmol [0.05–0.33]; *p* = 0.0001) and healthy controls (0.28 μg/mmol [0.10–0.51]; *p* = 0.002) (Fig. 2C). No other significant differences between the groups were noted. Urinary complement C5a concentration was statistically significantly increased in IgAV-N patients (101.9 ng/mmol [15.36–230.0]) compared to patients grouped as IgAVwoN (18.33 ng/mmol [4.27–33.3]; *p* = 0.01), and all other groups – SLEwoLN (22.81 ng/mmol [3.92–30.79]; *p* = 0.03), LN (11.28 ng/mmol [6.14–16.88]; *p* = 0.0004) and healthy controls (14.99 ng/mmol [3.19–29.31]; *p* = 0.002) (Fig. 2D). No other significant differences between the groups were noted. Interestingly, there were notable outliers seen within the IgAV subgroups (as illustrated in Fig. 2) and these may suggest that certain patients within the nephritis group express a more pronounced complement protein profile.

### Urinary complement concentrations distinguishing patients with IgAV-N

**Fig. 3:**
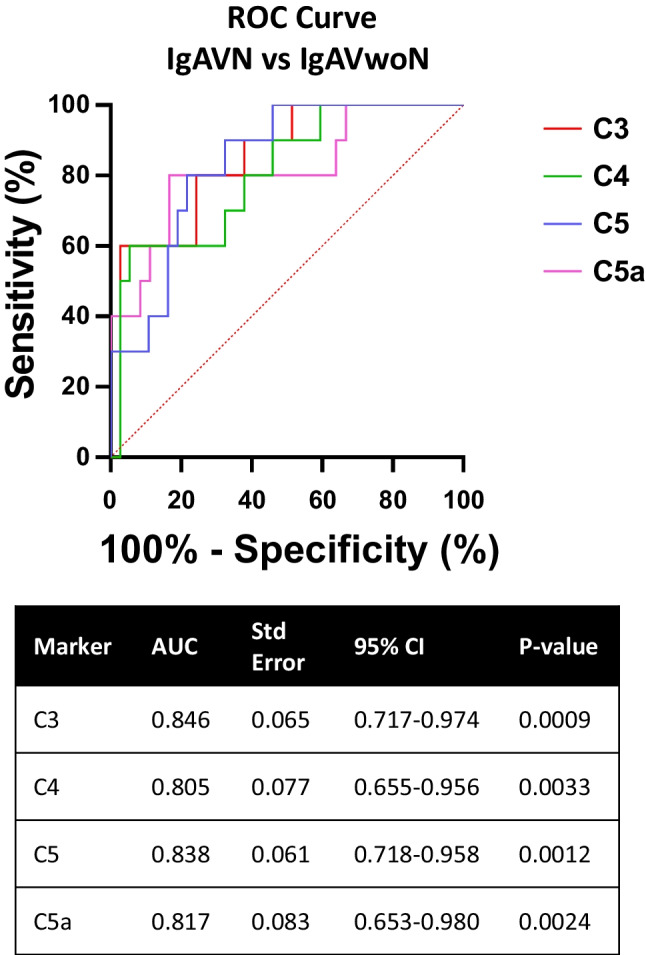
Receiver Operating Characteristic (ROC) curve analysis for IgAV-N patient individual complement concentrations compared to IGAVwoN. Urinary complement concentrations were analysed individually for their sensitivity and 100%—specificity in order to determine their effectiveness at distinguishing between IgAV-N patients vs. IgAVwoN patients

**Fig. 4:**
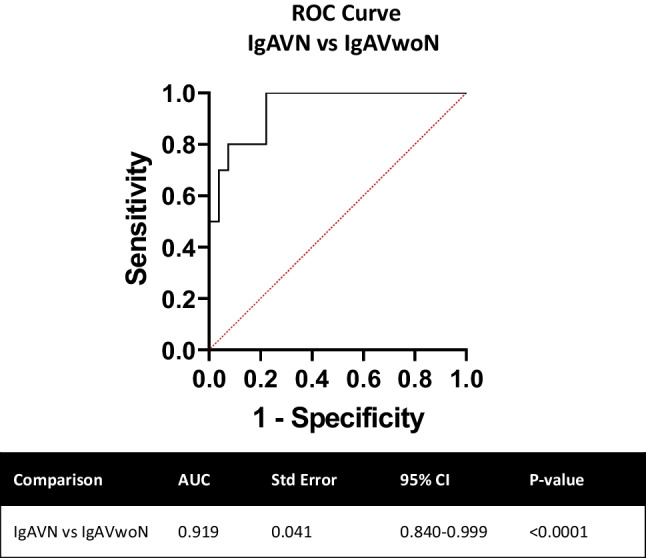
Receiver Operating Characteristic (ROC) curve analysis for IgAV-N patient combined complement concentrations compared to IGAVwoN. Urinary complement concentrations were analysed together using logistic regression analysis for their sensitivity and 100%—specificity in order to determine their effectiveness at distinguishing between IgAV-N patients vs. IgAVwoN patients

Receiver operating characteristic (ROC) curve analyses were used to determine the ability of the complement proteins to discriminate between patients with IgAV-N and those without nephritis. The level of each urinary complement molecule (C3, C4, C5, C5a) was individually excellent at distinguishing between patients with IgAV-N and IgAVwoN (AUC; C3 – 0.085, *p* = 0.0009; C4 – 0.805, *p* = 0.0033; C5 – 0.838, *p* = 0.0012; and C5a – 0.817, *p* = 0.0024) (Fig. 3). Logistic regression analysis was used to combine all four complement markers into a single biomarker panel and ROC analysis was performed. Combining all four urinary complement components (C3, C4, C5 and C5a) improved the effectiveness of the test to outstanding (AUC; IgAV-N vs. IgAVwoN – 0.919, *p* < 0.001) (Fig. 4).

## Discussion

IgAV is a common paediatric condition and the second most frequent reason to perform a kidney biopsy by paediatric nephrologists. It is well recognised that kidney outcomes from IgAV have failed to improve over time and current treatments lack robust evidence to support their use. Due to growing interest in the role of complement in IgA nephropathy, a condition histologically similar to IgAV, this study aimed to evaluate whether urinary complement proteins can be measured in children with IgAV, how they compare to another form of glomerulonephritis, and if they may indicate the presence of nephritis. This study demonstrated that urinary complement C3, C4, C5, and C5a concentrations were all statistically significantly increased in children with IgAV-N. Each urinary complement marker was individually excellent at distinguishing between those with and without nephritis and when combined, using a logistic regression analysis, their ability to discriminate between children with IgAV with or without nephritis was outstanding (AUC – 0.919).

Histologically, evidence of complement deposition is well recognised in IgAV-N, with approximately 74% of patients having histological evidence of C3 deposition identified in studies [10]. The complement system is important in linking the innate and adaptive immunity and it can be activated by different routes, known as the classical pathway, mannose-binding lectin pathway and alternative pathway. These specific pathways lead to a common pathway and the production of the membrane attack complex (C5b-9) causing cell lysis, opsonisation and upregulation of C5a, a neutrophil chemoattractant protein. Evidence of activation of the mannose-binding lectin pathway and/or the alternative complement pathway is reported in the skin and systemically in patients with IgAV [14, 15]. To our knowledge, this is the first report of measuring urinary complement in children with IgAV-N. Previous reports on other proteinuric conditions demonstrate that components of the complement system are able to distinguish between patients with membranous nephropathy and diabetic nephropathy independently of the amount of protein loss [7]. In patients with SLE, systemic complement abnormalities are well described but their precise role is not clearly understood, and surprisingly there are few studies measuring urinary complement in patients with LN [16]. Elevated urinary expression of complement is not found in patients with minimal change nephrotic syndrome suggesting that their presence doesn’t seem to be directly related to heavy proteinuria [9]. This study highlights important findings as urinary complement may represent a reliable biomarker in an easily accessible biofluid to stratify patients with IgAV for potential early therapeutic intervention that may mitigate the onset of nephritis. The clear outliers with increased complement expression highlighted in this study give the impression that there may be certain individuals with nephritis who would benefit the most from this intervention. While urine complement products are not routinely measured in clinical laboratories at present, these can be measured in the pre-clinical and pharmaceutical setting and further studies are required to understand the specific role of complement pathway in the pathogenesis of IgAV-N. As the complement pathway is a target of multiple new therapeutic agents currently under evaluation in clinical trials for many inflammatory kidney diseases, gaining insight into their role in IgAV may provide evidence for broader use of these medications [17].

This study does have limitations. Notably, there were assumptions in presuming a negative urine dipstick represented no proteinuria and therefore microalbuminuria may have been missed. Additionally, it did not demonstrate any significant increase in the urinary complement proteins in children with LN, despite the recognised role that complement has in this disease [18]. This may represent limitations in selecting the LN cohort who were defined as having nephritis according to the kidney domain of the BILAG 2004, and patients with chronic residual proteinuria may have been included, a known limitation of the SLE disease activity index [19]. This is supported by the relatively low concentration of urinary protein seen within the LN cohort, which is unusual in acute LN, and the long time elapsed since diagnosis, suggesting that they may have reflected a cohort of children with previous LN and persisting proteinuria. Other limitations of this study include the cross-sectional nature of the study and the relatively small, heterogeneous cohort of patients that may have limited the generalisability of the findings. It would be difficult to determine whether the urinary complement concentrations were merely increased due to the finding of proteinuria, as patients with nephritis were defined according to the presence of proteinuria and recent reports suggest a complex interplay between proteinuria and its potential to upregulate the tubular expression of complement pathway products [8]. Despite these limitations, our exploratory findings report that urinary complement products are measurable in children with IgAV and our data support a potential role for complement as a contributor to the pathophysiology of IgAV-N. Additional large, longitudinal studies are required to observe a large cohort of children from disease presentation to improvement or the evolution of nephritis to determine the timing of when complement may have its onset in the disease process and to identify when complement could be inhibited as a potential therapeutic target.

## Supplementary Information

Below is the link to the electronic supplementary material.

Graphical Abstract (PPTX 107 KB)
