# Supplementary material for: Urinary complement proteins are increased in children with IgA vasculitis (Henoch-Schönlein purpura) nephritis
Source: Pediatr Nephrol. 2022 Oct 13;38(5):1491–8. doi: 10.1007/s00467-022-05747-3 (PMC10060309; doi:10.1007/s00467-022-05747-3)
Supplement: Supplementary file 1 — Graphical Abstract (PPTX 107 KB) [file 467_2022_5747_MOESM1_ESM.pptx]

## Slide 1
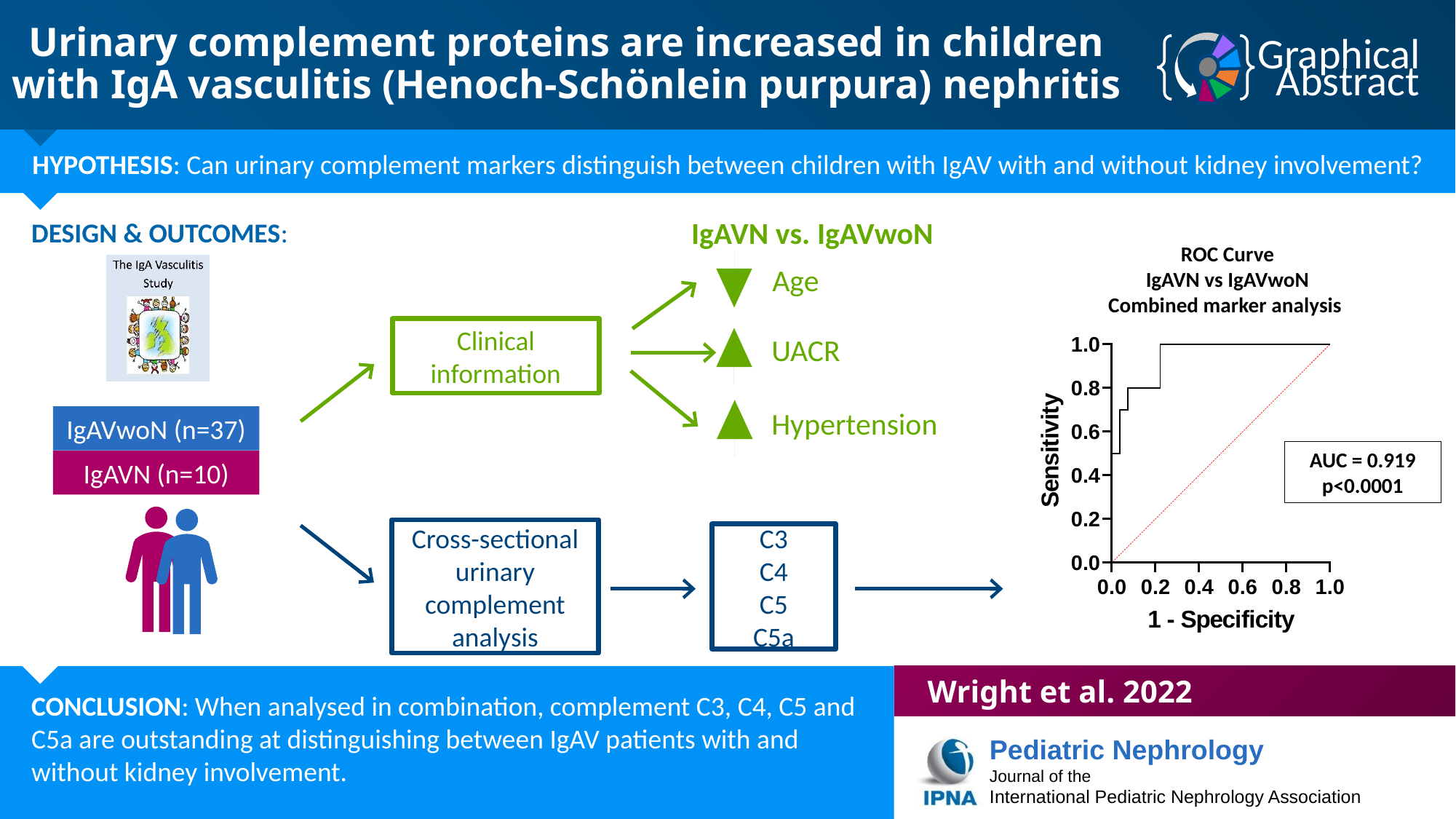

Urinary complement proteins are increased in children with IgA vasculitis (Henoch-Schönlein purpura) nephritis
HYPOTHESIS: Can urinary complement markers distinguish between children with IgAV with and without kidney involvement?
IgAVN vs. IgAVwoN
DESIGN & OUTCOMES:
ROC Curve
IgAVN vs IgAVwoN
Combined marker analysis
AUC = 0.919
p<0.0001
Age
Clinical information
UACR
Hypertension
IgAVwoN (n=37)
IgAVN (n=10)
Cross-sectional urinary complement analysis
C3
C4
C5
C5a
Wright et al. 2022
CONCLUSION: When analysed in combination, complement C3, C4, C5 and C5a are outstanding at distinguishing between IgAV patients with and without kidney involvement.
